# Wnt/β-catenin signaling, which is activated in odontomas, reduces Sema3A expression to regulate odontogenic epithelial cell proliferation and tooth germ development

**DOI:** 10.1038/s41598-019-39686-1

**Published:** 2019-03-12

**Authors:** Shinsuke Fujii, Kengo Nagata, Shinji Matsumoto, Ken-ichi Kohashi, Akira Kikuchi, Yoshinao Oda, Tamotsu Kiyoshima, Naohisa Wada

**Affiliations:** 1 Laboratory of Oral Pathology, Division of Maxillofacial Diagnostic and Surgical Sciences, Faculty of Dental Science, Kyushu University, 3-1-1 Maidashi, Higashi-ku, Fukuoka, 812-8582 Japan; 2 Department of Molecular Biology and Biochemistry, Graduate School of Medicine, Osaka University, 2-2 Yamadaoka, Suita, 565-0871 Japan; 3 Department of Anatomic Pathology, Graduate School of Medical Sciences, Kyushu University, 3-1-1 Maidashi, Higashi-ku, Fukuoka, 812-8582 Japan; 4 Division of General Dentistry, Kyushu University Hospital, Kyushu University, 3-1-1 Maidashi, Higashi-ku, Fukuoka, 812-8582 Japan

## Abstract

Odontomas, developmental anomalies of tooth germ, frequently occur in familial adenomatous polyposis patients with activated Wnt/β-catenin signaling. However, roles of Wnt/β-catenin signaling in odontomas or odontogenic cells are unclear. Herein, we investigated β-catenin expression in odontomas and functions of Wnt/β-catenin signaling in tooth germ development. β-catenin frequently accumulated in nucleus and/or cellular cytoplasm of odontogenic epithelial cells in human odontoma specimens, immunohistochemically. Wnt/β-catenin signaling inhibited odontogenic epithelial cell proliferation in both cell line and tooth germ development, while inducing immature epithelial bud formation. We identified Semaphorin 3A (Sema3A) as a downstream molecule of Wnt/β-catenin signaling and showed that Wnt/β-catenin signaling-dependent reduction of Sema3A expression resulted in suppressed odontogenic epithelial cell proliferation. Sema3A expression is required in appropriate epithelial budding morphogenesis. These results suggest that Wnt/β-catenin signaling negatively regulates odontogenic epithelial cell proliferation and tooth germ development through decreased-Sema3A expression, and aberrant activation of Wnt/β-catenin signaling may associate with odontoma formation.

## Introduction

Odontomas are classified as odontogenic benign tumors, comprising odontogenic epithelium and odontogenic ectomesenchyme with disorganized dental hard tissue formation in the World Health Organization (WHO) Classification of Head and Neck Tumours^1^; these are thought to be developmental anomalies of tooth germ, such as hamartomas, rather than benign neoplasms. Odontomas are the most common odontogenic tumors, with an incidence of 0.24–1.24%^2^. Although several possible factors are shown to be involved in odontoma development (e.g., heredity, genetic mutations and trauma during primary dentition)^3^, definitive mechanisms in the induction of odontomas remain to be clarified. In particular, it remains unclear whether any growth factor signalings are involved in odontoma development to date.

Tooth formation is initiated by tooth germ development and involves continuous and sequential steps, which are regulated by reciprocal interactions between odontogenic epithelium and adjacent mesenchyme^4,5^. Signalings related to several growth factors, such as Wnt, bone morphogenetic protein (BMP), fibroblast growth factor (FGF) and sonic hedgehog (SHH), have been reported to be essential in its development^4,5^. In studies with genetically modified mice, Wnt signaling was revealed to be necessary and sufficient for tooth germ development^6–8^, but the underlying molecular mechanism for Wnt-regulated tooth germ development remains unclear.

Familial adenomatous polyposis (FAP) and Gardner’s syndrome, a phenotypic variant of FAP, are an autosomal dominant cancer predisposition syndrome caused by *adenomatous polyposis coli* (*APC*) gene mutation and the patients exhibit intestinal polyposis, resulting in malignant tumors^9–13^. Of note, dental anomalies, such as odontomas, are present in 30–75% of Gardner’s syndrome patients and 9.4-83.3% of patients with FAP^14,15^. Wnt signaling regulates two different pathways: the β-catenin pathway and the β-catenin-independent pathway. In the former pathway, APC organizes a multiprotein “destruction complex” that degrades β-catenin, and Wnt signaling induces gene expression through the inhibition of “destruction complex” and β-catenin stabilization^16^. Therefore, loss-of-function of APC found in FAP or Gardner’s syndrome leads to the activation of the β-catenin pathway for regulating gene expression, which may promote odontomas.

Consistent with other studies^6–8,17^, conditional gain-of-function of the β-catenin pathway in keratin14-positive odontogenic epithelium, even in postnatal mice, resulted in supernumerary teeth resembling odontomas, suggesting that ectopic activation of the β-catenin pathway in odontogenic epithelium might be involved in odontomas^18^. It was also reported that activation of the β-catenin pathway in SOX2-positive cells, which were thought to be odontogenic epithelial stem cells^19,20^, was involved in odontomas^21^. Although these studies revealed the importance of the β-catenin pathway in developmental anomalies such as odontomas, the precise function of the β-catenin pathway in odontogenic epithelial cells or in tooth germ development remains unclear. Herein, we investigated the activation of the β-catenin pathway in the remaining epithelial cells within human odontomas and the function of its pathway to control cellular growth through regulating gene expression in both odontogenic epithelial cells and tooth germ development.

## Results

### Expression of β-catenin in odontomas

**Figure 1:**
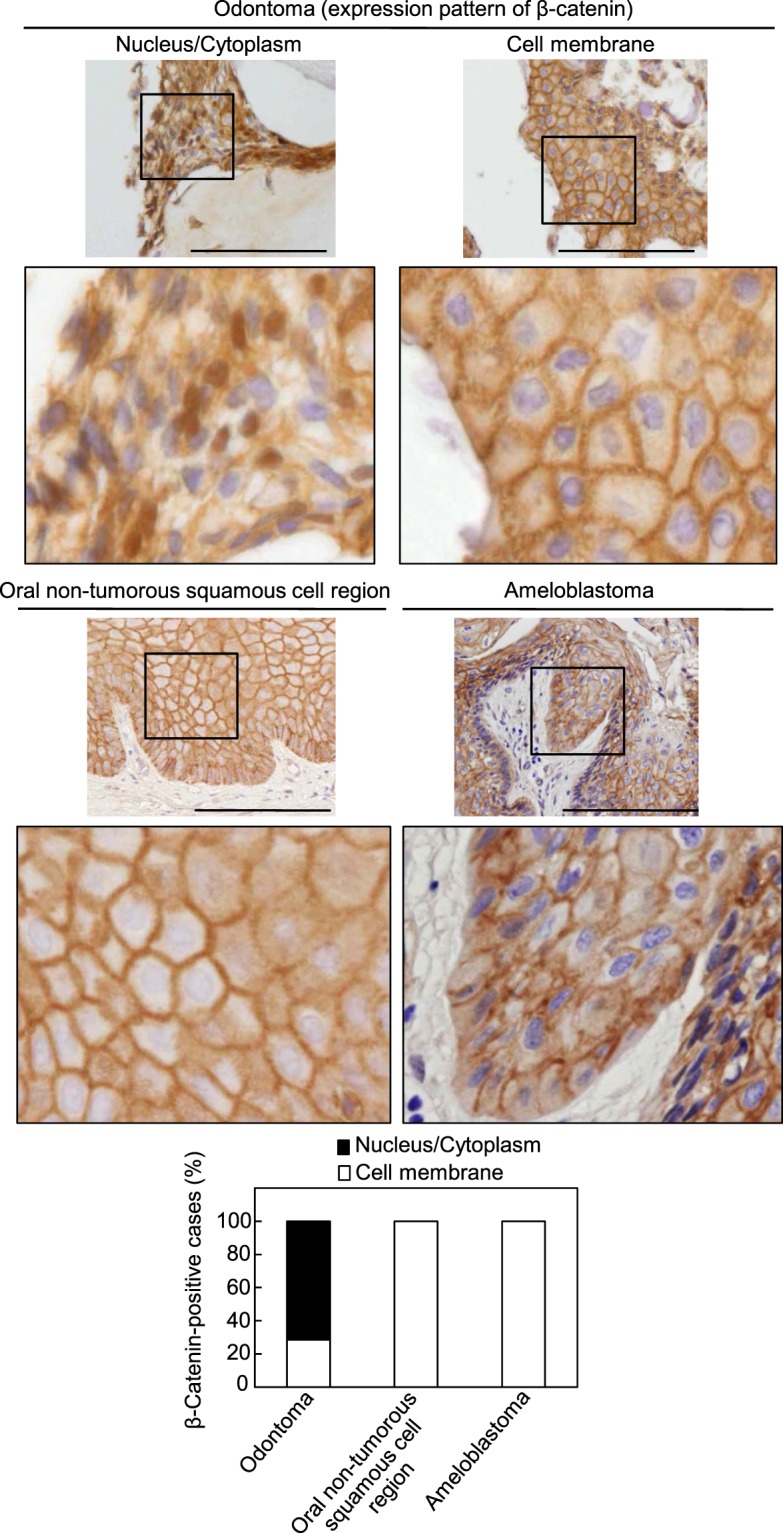
Expression of β-catenin in the remaining epithelial cells within human odontomas. Odontoma tissues (*n* = 21), oral non-tumorous stratified squamous cell regions (*n* = 9) and ameloblastomas (*n* = 6) were stained with anti-β-catenin antibody and hematoxylin. The areas stained with β-catenin were classified into two categories: nucleus and/or cellular cytoplasm (nucleus/cytoplasm) or cell membrane. Percentages of β-catenin-positive cases in each category of the odontomas, oral non-tumorous stratified squamous cell regions and ameloblastomas are shown in the lower panel. Black boxes show enlarged images. Scale bars: 100 μm.

To examine whether the β-catenin pathway is activated in odontomas, the expression pattern of β-catenin was investigated in the remaining odontogenic epithelial cells within human odontomas specimens by using immunohistochemical analyses. The areas stained with β-catenin were classified into two categories: nucleus and/or cellular cytoplasm (nucleus/cytoplasm) or cell membrane (Fig. 1). The results were considered positive and categorized in above classification when >50% of the total epithelial cells showed a similar pattern within a single specimen. In cases of human odontomas, β-catenin had accumulated in the nucleus/cytoplasm of odontogenic epithelial cells in 15/21 (71.4%) cases and in the cell membrane of odontogenic epithelial cells in 6/21 (28.6%) cases. In contrast, β-catenin was detected in the cell membrane of squamous cells of the oral non-tumorous stratified squamous cell region in 9/9 (100%) cases and in the odontogenic epithelial cells of ameloblastomas in 6/6 (100%) cases, suggesting that the β-catenin pathway is specifically activated in odontomas. In odontomas, odontogenic epithelial cells were found in islet or cord forms within immature connective tissue. β-catenin was more frequently accumulated in the nucleus/cytoplasm in islets of remaining odontogenic epithelial cells than in cords (Fig. S1a and Table 1), indicating that the β-catenin pathway in the remaining odontogenic epithelial cells is likely to be activated in the islet form. In addition, odontomas are subdivided into compound or complex type^1^, and positive rates of β-catenin staining were not significantly different between the types (Table 2). To elucidate whether the activation of the β-catenin pathway was associated with genetic mutations of the *CTNNB1* (β*-catenin*) gene or the *adenomatous polyposis coli* (*APC*) gene, genomic DNA from the two odontoma specimens with accumulation of β-catenin in nucleus/cytoplasm pattern (Fig. 1, left panels and Fig. S1b, left panels) was subjected to direct sequencing of exon 3 of the *CTNNB1* gene, or of exon 15 (from codons 1274 to 1523) of the *APC* gene. However, no mutations of *CTNNB1* (Fig. S1b, right panel) or *APC* (data not shown) were detectable in either of these specimens, suggesting that the activation of the β-catenin pathway might not depend on genetic mutations in these two odontomas.

**Table 1:** Relationship between β-catenin expression and epithelial patterns in cases of human odontoma (n = 21).

β-Catenin expression			
	Nucleus + Nucleus/Cytoplasm	Cell membrane	*P* value
Epithelial patterns			
Islets	12 (85.7%)	2	0.0404
Cords	3 (42.9%)	4

**Table 2:** Relationship between β-catenin expression and classification of human odontoma (n = 21).

β-Catenin expression			
	Nucleus + Nucleus/Cytoplasm	Cell membrane	*P* value
Classification of odontomas			
Compound	12 (80%)	3	0.1692
Complex	3 (50%)	3	

### Sema3A is a downstream molecule of the β-catenin signaling in odontogenic epithelial cells

**Figure 2:**
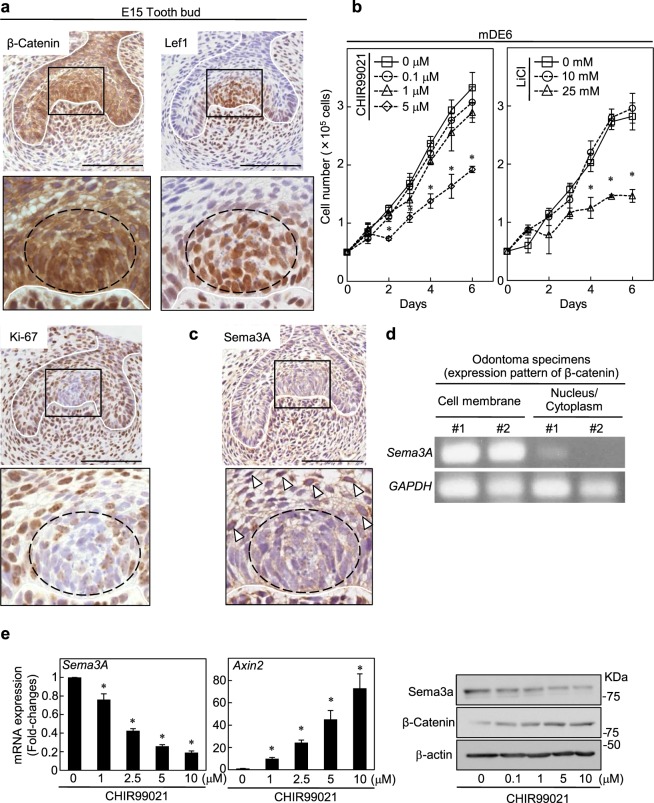
Sema3A is a downstream molecule of the β-catenin signaling in odontogenic epithelial cells. (**a**) Tissue sections of mouse tooth germ at E15 were stained with anti-β-catenin (upper left panels), anti-Lef1 (upper right panels) or anti-Ki-67antibody (lower panels) and hematoxylin. Black boxes show enlarged images. Dotted lines and white lines indicate the enamel knot region and the border between odontogenic epithelium and mesenchyme, respectively. (**b**) mDE6 cells were cultured without or with 0.1, 1 and 5 μM CHIR99021 or with 10 and 25 mM LiCl in the presence of 2% serum for the indicated numbers of days, and cell numbers were counted. (**c**) Tissue sections of mouse tooth germ at E15 were stained with anti-Sema3A and hematoxylin. Black boxe shows an enlarged image. Arrowheads indicate Sema3A-positive cells. Dotted line and white line indicate the enamel knot region and the border between odontogenic epithelium and mesenchyme, respectively. (**d**) Purified RNAs from human odontoma specimens were subjected to PCR and the products were loaded on agarose gel. (**e**) Expression levels of *Sema3A* or *Axin2* mRNA in mDE6 cells, which were cultured without or with 1, 2.5, 5 and 10 μM CHIR99021 for 24 h, were measured and expressed as fold-changes compared with levels in control cells (left two graphs). mDE6 cells were cultured without or with 0.1, 1, 5 and 10 μM CHIR99021 for 24 h, and then cell lysates were probed with anti-Sema3A, anti-β-catenin or anti-β-actin antibody (right panel). Results are shown as means ± s.d. of three independent experiments. *p < 0.01. Scale bars: 100 μm (**a**,**c**).

β-catenin has been reported to be accumulated in the tooth germ enamel knot region, a signaling center in which several growth factor signaling pathways are activated; this region is known to regulate the growth and differentiation of the enamel organ^18,22^. Consistent with these reports, immunohistochemical analysis revealed accumulation of β-catenin and expression of lymphoid enhancer binding factor 1 (Lef1, a target gene of the Wnt/β-catenin pathway) in the tooth germ enamel knot regions in mouse at E15; Ki-67 was not detectable (Figs 2a and S2a). These results indicated that the β-catenin pathway is specifically activated in the enamel knot region to regulate the proliferation of enamel knot epithelial cells negatively.

To examine the roles of the β-catenin pathway in odontogenic epithelial cells, mDE6 (mouse odontogenic epithelial cells) were treated with the GSK-3 inhibitor, CHIR99021 or LiCl, which is an activator of the β-catenin pathway^23,24^. Both CHIR99021 and LiCl suppressed mDE6 cell proliferation capability in a dose-dependent manner (Fig. 2b), suggesting that the β-catenin pathway might function as a negative regulator of odontogenic epithelial cellular growth. Consistently, CHIR99021 treatment did not induced *cyclin D1* mRNA expression (Fig. S2b), which is a target gene of the β-catenin pathway to induce cellular proliferation capability, indicating that other β-catenin pathway target genes may regulate cellular proliferation. To detect target genes mediating antiproliferative effect of the β-catenin pathway, DNA microarray analysis of mDE6 cells with 6 h stimulation of CHIR99021 was performed. Candidate genes were selected based on the criterion that their expression levels were lower in cells treated with CHIR99021 than in the control cells. In addition, functional annotation clustering was carried out by using the DAVID database (http://david.abcc.ncifcrf.gov/). Among possible candidate genes, Semaphorin 3A (Sema3A), which belongs to the semaphorin family, was selected for further analysis. Sema3A expression was clearly decreased in DNA microarray data and the DAVID database revealed that Sema3A was a member of several clusters, such as developmental protein, multicellular organism and differentiation (Table S1). Sema3A was not a member of the cluster of regulation of cell growth; however it was recently reported that Sema3A is involved in cell proliferation in both glioma and glioblastoma cells^25,26^. While crosstalk between Sema3A signaling and the β-catenin pathway has been shown in osteoblasts^27^, the function of Sema3A in odontogenic epithelial cells is not yet understood. It is noteworthy that Sema3A expression was suppressed specifically in enamel knot region (Fig. 2c), where the β-catenin pathway is activated, immunohistochemically. Both Sema3A and Ki-67 were co-expressed in stellate reticulum cells around the enamel knot (Fig. 2a,c). Stellate reticulum cells are likely to act as a cushion against physical forces during tooth development^28^ and enamel epithelial stem cell-like cells are included in them^29^. Moreover, *Sema3A* mRNA expression was lower in specimens which β-catenin was accumulated in the nucleus/cytoplasm than in those with β-catenin-accumulated cell membrane (Fig. 2d). Therefore, in the following study, the expression mechanism and function of Sema3A was examined.

Quantitative RT-PCR demonstrated that mDE6 cells showed higher expression of *Sema3A* mRNA than mouse odontogenic mesenchymal cells (mDP)^30^, but lower expression than tooth germ at embryonic day (E) 15 (Fig. S2c). Consistent with DNA microarray analysis, we confirmed that CHIR99021 dramatically reduced Sema3A mRNA and protein levels, but promoted *Axin2* (a direct target gene of the Wnt/β-catenin pathway) mRNA expression or the accumulation of β-catenin in a dose-dependent manner in mDE6 cells (Fig. 2e). Concomitantly, stimulation of mDE6 cells with Wnt3a or LiCl resulted in similar effects on their gene expression (Fig. S2d). In addition, immunofluorescence data revealed that β-catenin was accumulated in the nucleus and that cytoplasmic Sema3A expression was reduced by CHIR99021 treatment in mDE6 cells (Fig. S2e). These results indicate that Sema3A is a possible downstream gene of Wnt/β-catenin signaling.

### Activation of the β-catenin pathway suppresses the proliferation of odontogenic epithelial cells through a decrease of Sema3A expression

**Figure 3:**
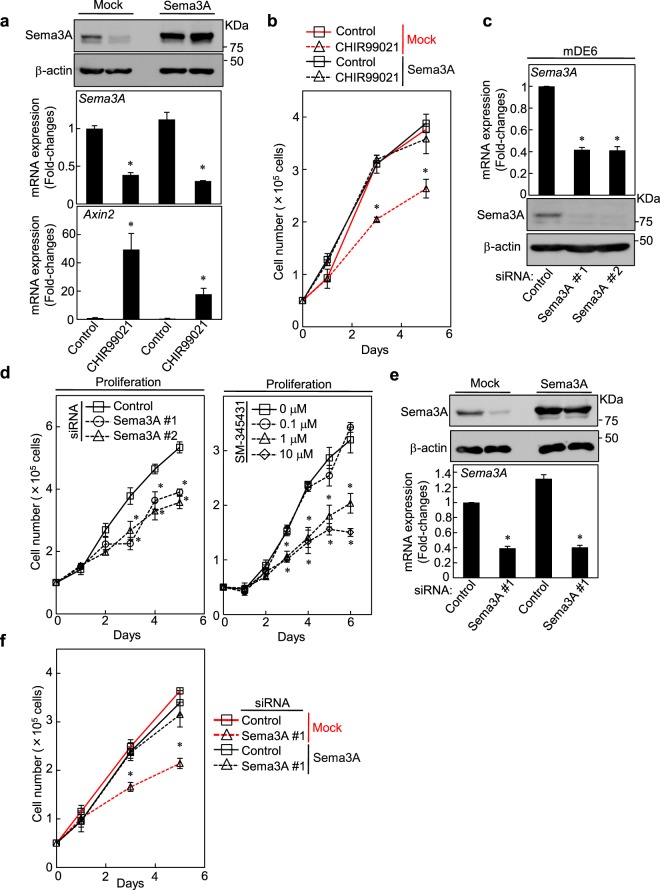
Activation of the β-catenin pathway suppresses the proliferation of odontogenic epithelial cells through a decrease of Sema3A expression. (**a**) mDE6 cells expressing mock or Sema3A were cultured without or with 5 μM CHIR99021 for 24 h. Cell lysates were probed with anti-Sema3A and anti-β-actin antibodies. *Sema3A* or *Axin2* mRNA levels were measured by quantitative RT-PCR using primers encoding the 3′-UTR (for *Sema3A*). Relative *Sema3A* or *Axin2* mRNA expression levels were normalized by *GAPDH* and expressed as fold-changes compared with levels in control mock cells. (**b**) mDE6 cells expressing mock or Sema3A were cultured without or with 5 μM CHIR99021 in the presence of 2% serum for the indicated numbers of days, and cell numbers were counted. (**c**) mDE6 cells were transfected with control or two independent Sema3A siRNAs, and *Sema3A* mRNA levels were measured by quantitative RT-PCR. Relative *Sema3A* mRNA levels were normalized by *GAPDH* and expressed as fold-changes compared with levels in control siRNA transfected cells. Cell lysates were probed with anti-Sema3A and anti-β-actin antibodies. (**d**) mDE6 cells were transfected with control or two independent Sema3A siRNAs (left panel), or were cultured without or with 0.1, 1 and 10 μM SM-345431 (right panel) in the presence of 2% serum for the indicated numbers of days, and cell numbers were counted. (**e**,**f**) mDE6 cells expressing mock or Sema3A were transfected with control or Sema3A #1 siRNA. (**e**) Cell lysates were probed with anti-Sema3A and anti-β-actin antibodies. *Sema3A* mRNA levels were measured by quantitative RT-PCR using primers encoding the 3′-UTR. Relative *Sema3A* mRNA levels were normalized by *GAPDH* and expressed as fold-changes compared with levels in control siRNA transfected mock cells. (**f**) The cells were cultured in the presence of 2% serum for the indicated numbers of days, and cell numbers were counted. Results are shown as means ± s.d. of three independent experiments. *p < 0.01.

Lentivial transduction with Sema3A rescued the CHIR99021-dependent reduction of Sema3A protein expression (Fig. 3a). Meanwhile, in mDE6 expressing cells mock or Sema3A, CHIR99021 decreased endogeneous *Sema3A* mRNA expression using primers encoding 3′-UTR (untranslated region) (Fig. 3a). Exogenous Sema3A expression did not affect CHIR99021-dependent cytoplasmic accumulation and nuclear translocation of β-catenin, but decreased *Axin2* mRNA expression (Fig. S3a,b). Importantly, exogenous Sema3A expression rescued the CHIR99021-dependent inhibitory effect on cellular growth (Fig. 3b). These results suggest that the activation of the β-catenin pathway results in the suppression of Sema3A expression to regulate odontogenic epithelial cell proliferation.

To elucidate the roles of Sema3A in mDE6 cells, Sema3A was knocked down by two different siRNAs (Fig. 3c). Sema3A knock down did not affect activation of AKT (S473) and ERK1/2 (T202/Y204), while increasing *Axin2* mRNA levels slightly, but significantly (Fig. S3c). Sema3A knockdown decreased proliferation and migration of mDE6 cells (Fig. 3d, left and Figure. S3d); SM-345431, which functions as a Sema3A inhibitor through diminishing Sema3A binding to its receptor neuropilin-1^31^, also reduced mDE6 cell proliferation (Fig. 3d, right). These results suggest that endogenous Sema3A-neuropilin-1 signaling regulates cell proliferation. To further examine the roles of Sema3A, mDE6 cells expressing mock or Sema3A were transfected with control or Sema3A #1 siRNA. Lentivial transduction with Sema3A rescued siRNA-dependent reduction of Sema3A protein expression (Fig. 3e). As we used siRNAs that target the 3′-UTR (see Table S2), they did not decrease the amount of exogenously expressed Sema3A in the cells. The effect of Sema3A #1 siRNA was confirmed by quantitative RT-PCR, which showed that endogenous *Sema3A* mRNA levels were indeed decreased by Sema3A #1 siRNA using primers encoding 3′-UTR (Fig. 3e). Cells expressing Sema3A showed the similar proliferation capability to control cells expressing mock (Fig. 3f), indicating that endogenous Sema3A is sufficient for regulating the proliferative ability of mDE6 cells. Meanwhile, in both of cell proliferation assay and migration assay, exogenous Sema3A expression rescued the Sema3A-knockdown phenotypes of mDE6 cells, excluding siRNA off target effects (Figs 3f and S3e). These results suggest that Sema3A expression is involved in the proliferation and migration of odontogenic epithelial cells.

### Wnt signaling decreased Sema3A expression through the Wnt/β-catenin-Lef1 pathway

**Figure 4:**
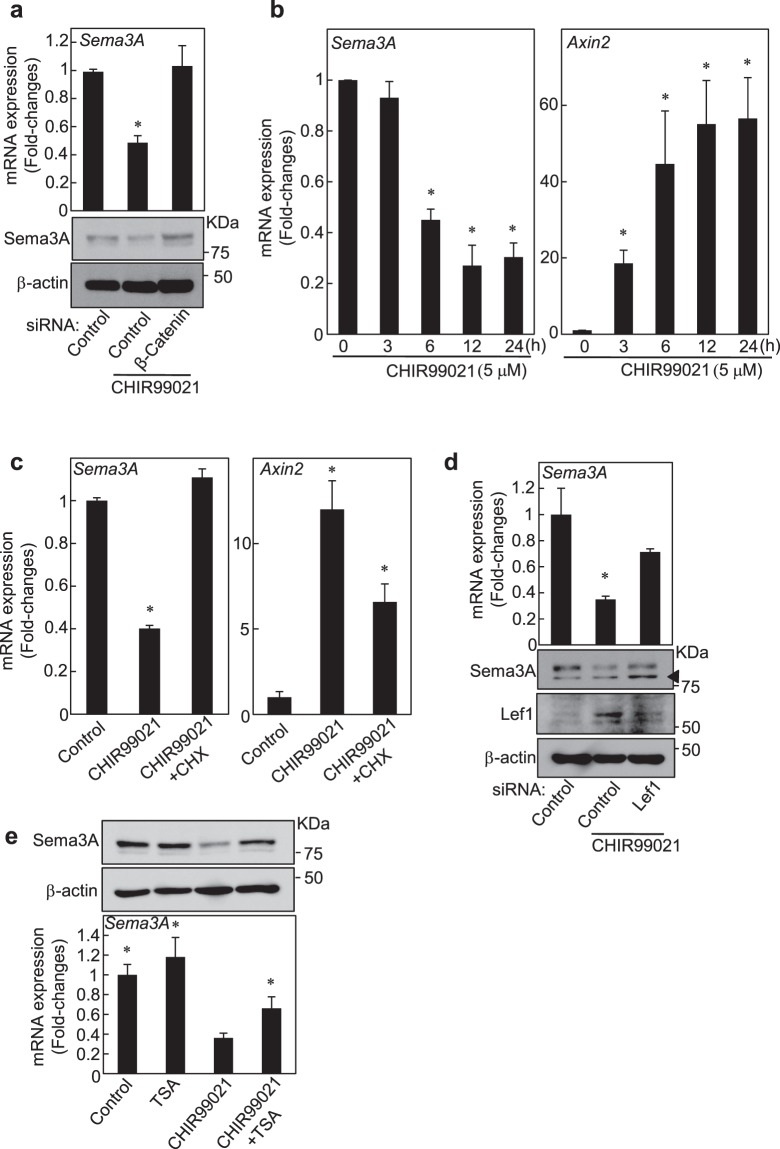
Wnt/β-catenin-Lef1 signaling downregulates Sema3A in odontogenic epithelial cells. (**a**) mDE6 cells were transfected with control or β-catenin siRNA, and then were cultured with 5 μM CHIR99021 for last 12 h. Relative *Sema3A* mRNA levels were normalized by *GAPDH* and expressed as fold-changes compared with levels in control siRNA transfected cells. Cell lysates were probed with anti-Sema3A and anti-β-actin antibodies. (**b**) Levels of *Sema3A* or *Axin2* mRNA in mDE6 cells, which were cultured with 5 μM CHIR99021 for 0, 3, 6, 12 or 24 h, were measured and expressed as fold-changes compared with levels in control cells. (**c**) mDE6 cells were untreated or treated with 5 μM CHIR99021 or a combination of 5 μM CHIR99021 and 0.1 μg/ml cycloheximide (CHX), and then *Sema3A* or *Axin2* mRNA expression was measured and expressed as fold-changes compared with levels in control cells. (**d**) mDE6 cells were transfected with control or Lef1 siRNA, and then were cultured with 10 μM CHIR99021 for last 12 h. Relative *Sema3A* mRNA levels were normalized by *GAPDH* and expressed as fold-changes compared with levels in control siRNA transfected cells. Cell lysates were probed with anti-Sema3A, anti-Lef1 or anti-β-actin antibody. Arrowhead indicates non-specific band. (**e**) mDE6 cells were treated without or with 5 μM CHIR99021 or a combination of 0.1 μM trichostatin A (TSA), and then *Sema3A* mRNA expression was measured and expressed as fold-changes compared with levels in CHIR99021-treated cells. Cell lysates were probed with anti-Sema3A and anti-β-actin antibodies. Results are shown as means ± s.d. of three independent experiments. *p < 0.01.

We examined the molecular mechanism by which CHIR99021 decreased Sema3A expression. Knockdown of β-catenin rescued CHIR99021-dependent down-regulation of Sema3A mRNA and protein expression, indicating that β-catenin is involved in its mechanism (Figs 4a and S4a). Treatment of mDE6 cells with CHIR99021 for 6, 12 and 24 h significantly decreased *Sema3A* mRNA expression; however, 3 h treatment did not, although *Axin2* mRNA expression was significantly higher than control even in 3 h treatment (Fig. 4b). Furthermore, treatment of the cells with cycloheximide (CHX), which inhibits protein synthesis, reversed CHIR99021-dependent down-regulation of *Sema3A* mRNA expression (Fig. 4c), suggesting that CHIR99021-dependent reduction of Sema3A expression is a result of protein synthesis. Lef1 transcription factor is a downstream target gene of the Wnt/β-catenin pathway^32,33^ and participates in the Wnt signaling pathway through the activation of target genes expression by binding to β-catenin^34^. Meanwhile, it is noteworthy that Lef1 also functions as a transcription repressor in osteoblasts, breast cancer cells and in leukemia cells through interactions with histone deacetylase-1 (HDAC-1)^35–37^. CHIR99021 treatment increased Lef1 mRNA and protein levels through β-catenin expression (Fig. S4b), and siRNA against Lef1 rescued CHIR99021-dependent down-regulation of Sema3A mRNA and protein expression (Figs 4d and S4c). In addition, treatment with trichostatin A (TSA), an HDAC inhibitor, partially rescued CHIR99021-dependent down-regulation of Sema3A mRNA and protein expression (Fig. 4e), indicating that histone modification might be involved in its mechanism. These data suggest that Wnt/β-catenin signaling induces Lef1 expression, thereby decreasing Sema3A expression in odontogenic epithelial cells.

### Activation of Wnt signaling disrupted tooth germ development through a decrease of Sema3A expression

**Figure 5:**
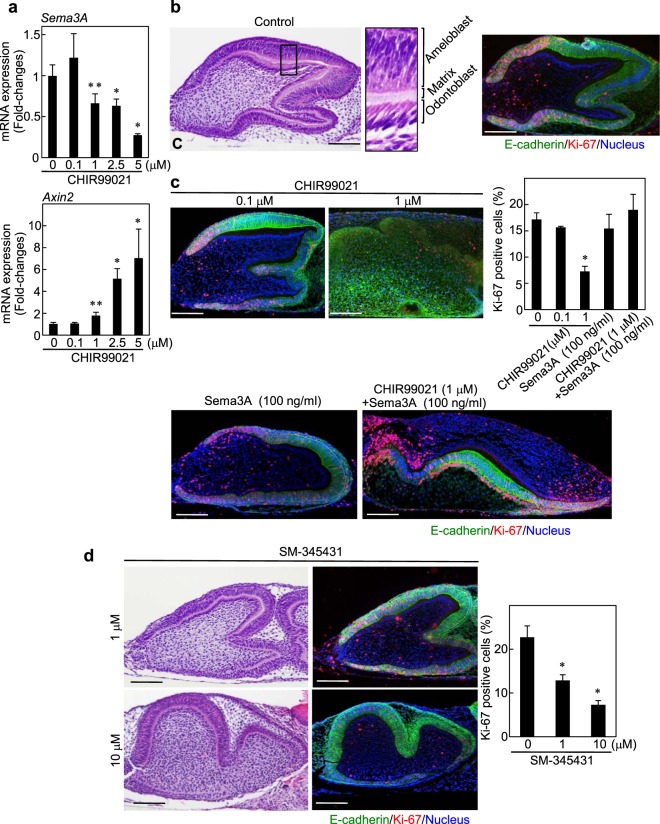
Wnt/β-catenin-Sema3A axis regulates epithelial cell proliferation in tooth germ development. (**a**) Levels of *Sema3A* or *Axin2* mRNA in tooth germ rudiments, which were treated without or with 0.1, 1, 2.5 and 5 μM CHIR99021 for 72 h, were measured and expressed as fold-changes compared with levels in control rudiments. (**b**) E15 tooth germ rudiments were cultured for 7 days, and then the rudiments were stained with hematoxylin and eosin (H&E). The rudiments were also stained with anti-E-cadherin and anti-Ki-67 antibodies, and Hoechst 33342. Black box shows an enlarged image. (**c**) Tooth germ rudiments were treated without or with 0.1 and 1 μM CHIR99021, 100 ng/ml Sema3A or 1 μM CHIR99021 with 100 ng/ml Sema3A for 7 days, and then the rudiments were stained with anti-E-cadherin and anti-Ki-67 antibodies, and Hoechst 33342. Ki-67-positive cells were counted, and results are expressed as the percentage of positively stained cells compared with total E-cadherin-stained cells (*n* = 19,196). (**d**) Tooth germ rudiments were cultured with 1 or 10 μM SM-345431 for 7 days, and then the rudiments were stained with H&E. The rudiments were also stained with anti-E-cadherin and anti-Ki-67 antibodies, and Hoechst 33342. Ki-67-positive cells were counted, and results are expressed as the percentage of positively stained cells compared with total E-cadherin-stained cells (*n* = 9,001). Results are shown as means ± s.d. of three independent experiments. *p < 0.01. **p < 0.05. Scale bars: 100 μm.

Finally, we evaluated the involvement of the Wnt/β-catenin-Sema3A axis in tooth germ development by using organ culture of tooth germ rudiments of E15 mouse embryos. Since E15 corresponds to late cap stage of mouse tooth germ development^38^, this organ culture is expected to mimic the developmental process from late cap stage to following developmental steps. In rudiments treated with CHIR99021 for 3 days, mRNA level of *Sema3A* was reduced, while mRNA level of *Axin2* was elevated in a dose-dependent manner (Fig. 5a). At day 7, control rudiments demonstrated thin matrix layers, which were interfaces between polarized ameloblasts and odontoblasts (Fig. 5b). In addition, Ki-67-positive cells were frequently observed in the cervical loops, mimicking tooth germ development *in vivo*^39^ (Fig. 5b). In rudiments treated with CHIR99021 for 7 days, the number of the proliferating epithelial cells, which were positive for both Ki-67 and E-cadherin, was decreased in a dose-dependent manner (Fig. 5c). Stimulation with Sema3A revealed a similar proliferation capability to control rudiments. Importantly, a 1 μM CHIR99021-dependent reduction of the number of proliferating epithelial cells was rescued by Sema3A stimulation (Figs 5c and S5). Furthermore, in the rudiments treated with SM-345431, the numbers of Ki-67-positive epithelial cells were clearly decreased (Fig. 5d).

**Figure 6:**
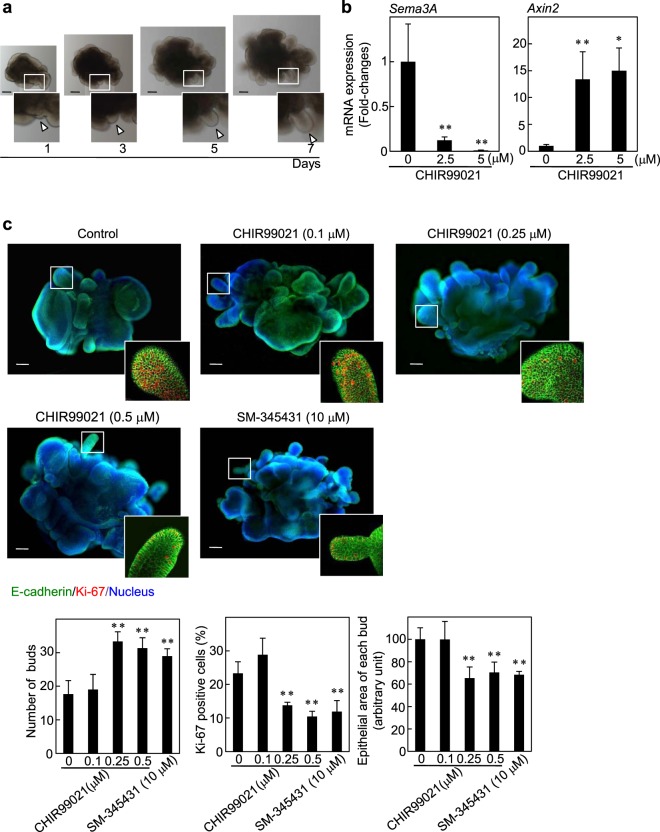
Involvement of Wnt/β-catenin-Sema3A axis in epithelial tooth bud culture. (**a**) Epithelial tooth buds isolated from E15 were cultured with FGF1 for 7 days in three-dimensional Matrigel. White boxes show enlarged images. Arrowheads indicate distal time-dependent protruded epithelia defined as “buds” in morphological. (**b**) Levels of *Sema3A* or *Axin2* mRNA in epithelial tooth buds, which were treated without or with 2.5 and 5 μM CHIR99021 for 72 h, were measured and expressed as fold-changes compared with levels in control epithelial tooth buds. (**c**) Epithelial tooth buds were cultured without or with 0.1, 0.25 and 0.5 μM CHIR99021 or 10 μM SM-345431 for 7 days, and then the epithelial tooth buds were stained with anti-E-cadherin and anti-Ki-67 antibodies, and Hoechst 33342. White boxes show enlarged images. The numbers of epithelial buds were counted (left graph). Ki-67-positive cells were counted, and results are expressed as the percentage of positively stained cells compared with total E-cadherin-stained cells (*n* = 3,909) (middle graph). The epithelial area of each bud was counted (right graph). Results are shown as means ± s.d. of three independent experiments. *p < 0.01. **p < 0.05. Scale bars: 100 μm.

To elucidate the involvement of the Wnt/β-catenin-Sema3A axis in tooth germ epithelial development, we established a mesenchyme-free epithelial tooth bud culture system from tooth germ rudiments of E15 mouse embryos. In this system, epithelial tooth buds underwent budding morphogenesis and distal time-dependent protruded epithelia were defined as “buds” in morphological (Fig. 6a). In the epithelial tooth buds treated with CHIR99021 for 3 days, mRNA expression of *Sema3A* was reduced, while mRNA expression of *Axin2* was elevated; these observations were consistent with our findings in tooth germ rudiments (Fig. 6b). At day 7, CHIR99021 treatment increased the number of buds, while reducing the number of the proliferating epithelial cells and each epithelial bud area, indicating that the induction of bud formation may not be a result of cell proliferation (Fig. 6c). In addition, SM-345431 treatment induced bud formation, while reducing the number of Ki-67-positive epithelial cells and each epithelial bud area (Fig. 6c). These data indicated that the activation of the β-catenin pathway suppresses Sema3A expression and epithelial cell proliferation, thereby leading to the induction of multiple bud structures and disruption of tooth germ development.

## Discussion

In this study, we demonstrated that β-catenin was accumulated in the nucleus/cytoplasm in 70% of the remaining odontogenic epithelial cells in human odontoma. We also found that the β-catenin pathway negatively regulates cellular growth through reduced Sema3A expression in odontogenic epithelial cells and its involvement in tooth germ development and in the immature epithelial tooth bud formation.

The mammalian tooth germ develops through the following four stages: the initiation, morphogenesis, cell differentiation and matrix secretion. Numerous signaling pathways are involved in the tooth germ developmental processes^5,40^. In the morphogenesis stage, the β-catenin pathway is spatiotemporally activated in the enamel knot region of tooth germ (see Fig. 2a); this region involves non-proliferative cells and regulates complex tooth shape development, such as grooves and tooth cusps^18,22^. Loss of FGF signaling due to reduced expression of FGF receptor and BMP-4 induced expression of cyclin-dependent kinase inhibitor p21 have been reported to be involved in the reduction of proliferative capabilities in enamel knot^38,41^. Based on our data, the Wnt/β-catenin pathway-dependent reduction of Sema3A expression might also be involved in the suppression of cell proliferation in the region, leading to proper tooth shape development.

Although odontomas are thought to be developmental anomalies of tooth germ, it is unclear whether Wnt/β-catenin signaling is involved in the etiology of odontomas. In the present study, β-catenin was expressed in the nucleus/cytoplasm with high frequencies in the remaining odontogenic epithelium of human sporadic odontoma specimens. Ectopic activation of the β-catenin pathway in the odontogenic epithelium of genetically modified mice resulted in an increase in the number of *shh*-positive-enamel knot regions, leading to the formation of well-mineralized but disorganized morphological supernumerary teeth resembling odontomas^18^. These data supports our immunohistochemical results in odontomas.

Genetic mutations of the *APC* or *Ctnnb1* gene were not detected in at least two current cases and the median age of the patients was 11 years old (see Methods); therefore, it is possible that another mechanism of β-catenin accumulation without genetic mutations may be present in odontomas.

To our knowledge, we have developed the first mesenchyme-free epithelial tooth bud culture system, similar to organoids of other organs, such as small intestine, kidney, salivary gland and lung^23,42–44^. Thickened epithelial tissue invaginates into underlying mesenchymal tissue during the initiation stage of tooth germ development; tooth morphogenesis then proceeds through sequential stages, such as bud, cap and bell stages^4,5^. It is intriguing to speculate that our system could be a model of the invagination and transition from bud, cap to bell stage. Importantly, the current epithelial tooth bud culture system revealed that the Wnt/β-catenin-Sema3A axis is involved in bud formation, and that the Wnt/β-catenin-Sema3A-dependent bud formation might be associated with disorganized tooth morphogenesis. Abnormal tooth germ development, such as epithelial smaller, ectopic invagination and multiple epithelial protrusions, was observed in genetically modified mice in which β-catenin pathway activation is dependent upon the odontogenic epithelium^8^. Furthermore, these tooth germs, which were transplanted into kidney capsules, exhibited supernumerary teeth similar to odontomas^7^, suggesting that regulated bud formation might be involved in tooth germ development. Collectively, aberrant ectopic activation of the β-catenin pathway during tooth germ developmental stages, though its mechanism remains unknown, could lead to the improper tooth development shown in odontomas, such as increased numbers and disorganized morphogenesis of tooth germ.

We identified Sema3A as a downstream gene of Wnt/β-catenin signaling. Lef1 knockdown rescued CHIR99021-dependent down-regulation of Sema3A mRNA and protein expression (See Figs 4d and S4c), demonstrating that Lef1 expression is required in its down-regulation. It has been reported that Lef1 functions as a transcription repressor in leukemia cells through interactions with histone deacetylase-1 (HDAC-1)^35^. Consistent with this report, treatment with TSA partially rescued CHIR99021-dependent down-regulation of Sema3A mRNA and protein expression (See Fig. 4e), therefore, it is possible that Lef1-mediated histone modification might be involved in its mechanism. Further study is needed to be clarified its precise mechanism.

Sema3A, which is a secreted protein, was originally identified as an axonal guidance factor that controls nerve system development in embryogenesis^45^. Sema3A and its receptors neuropilin signaling has been reported to be involved not only in cell proliferation or cell division in nerve system development, but also in the regenerative potential of dental tissue and tumorigenesis^46–48^. Since Sema3A-neuropilin signaling demonstrated positive effects on cell proliferation in glioma and glioblastoma^25,26^ and negative effect on cell proliferation in breast cancer^47,49^, its effects may depend on cell context. In odontogenic epithelial cells, loss-of-function experiments using siRNAs and inhibitor revealed that Sema3A expression is involved in their cell proliferation without activating of AKT and ERK1/2 signaling. As Sema3A inhibitor suppressed Ki-67-positive epithelial cell proliferation in tooth germ development and Sema3A stimulation rescued CHIR99021-dependent reduction of the number of proliferating epithelial cells, endogenous Sema3A expression and its signaling might be sufficient to regulate tooth germ development. Although the precise mechanism how Sema3A signaling regulate cellular growth remains unknown at present, expression of Sema3A might be involved in cellular proliferation of odontogenic epithelial cells. Further function of Sema3A in tooth development needs to be identified using mice with odontogenic epithelium-specific depletion of Sema3A in the future.

Sema3A positively regulate Wnt/β-catenin signaling in osteoblasts^27^, whereas osteosarcoma-derived Sema3A suppresses Wnt/β-catenin signaling^50^, suggesting that the effect of Sema3A on the Wnt/β-catenin signaling might depend on cell context. Consistent with the latter report, exogenous Sema3A expression decreased CHIR99021-dependent *Axin2* mRNA expression without affecting cytoplasmic accumulation and nuclear translocation of β-catenin and Sema3A knock down increased *Axin2* mRNA expression. It is possible that Sema3A signaling affects Wnt/β-catenin signaling at the transcriptional level in the nucleus, but its precise mechanism remains unclear. Taken together, it is indicated that Sema3A signaling suppresses Wnt/β-catenin signaling and vice versa in mDE6 cells.

## Methods

### Patients and odontoma tissues

Human odontoma (n = 40) and ameloblastoma (n = 6) tissues from patients, who underwent surgery at Kyushu University Hospital from January 2012 to April 2017 (total patients were 4,383), were examined in this study. Twenty one specimens of odontomas, which included odontogenic epithelium, were used for the immunohistochemical studies, and the ages of these patients ranged from 3 to 21 years (median, 11 years). All patients with odontoma were not diagnosed as FAP or Gardner’s syndrome, clinically. Odontomas were diagnosed according to the recent WHO Classification^1^. Consistent with previous reports^2^, the incidence of odontoma was 0.91% (40/4,383) in the present study. Resected specimens were macroscopically examined to determine the location and size of odontoma, and specimens for histology were fixed in 4% (w/v) paraformaldehyde (PFA) buffered by phosphate buffered saline (PBS) and decalcified in 10% (v/v) formic acid for 2 or 3 days, and processed for paraffin embedding. Specimens for examination were sectioned at 4 μm thickness and stained with hematoxylin and eosin for independent evaluations by three pathologists (K.N., S.F. and T.K.). The protocol for this study was approved by the ethical review board of the Local Ethical Committee of Kyushu University, Japan (#29–392), and the current study was performed in accordance with the committee guidelines and regulations. Informed consent was obtained from all patients.

### Immunohistochemical studies

Mouse embryos at E15 were collected and specimens were embedded in paraffin and sectioned at 5 μm thickness. Immunohistochemical studies for human odontomas and mouse tooth germ were performed as previously described^43,51^ with modification. For antigen retrieval, the sections were immersed in 10 mM trisodium citrate reagents for 10 min at 95 °C. The endogenous peroxidase activity was then eliminated by treatment with 1% hydrogen peroxide in methanol for 30 min. Non-specific protein binding was blocked with 10% normal goat serum (Nichirei, Tokyo, Japan) for 30 min. Tissue sections were incubated with anti-β-catenin (1:200), anti-Sema3A (1:600), anti-Ki-67 (1:300) or anti-Lef1 (1:300) antibody for 16 h at 4 °C, and then the sections were incubated with goat anti-mouse or anti-rabbit IgG-HRP (Histofine Simple Stain MAX PO, Nichirei) for 1 h at room temperature (RT). The immunoreactivity was visualized with a solution of 3,3′-diaminobenzidine and <0.1% hydrogen peroxide (Nichirei). Subsequently, the sections were counterstained with hematoxylin.

### Mouse tooth germ rudiment culture

Protocols used for all animal experiments in this study were approved by the Animal Research Committee of Kyushu University, Japan (No. A29-277-0), and the current study was conducted accordance to the institutional guidelines and regulations. As previously reported^52^, embryonic tooth germ (tooth germ rudiments) isolated from ICR mice at E15 were cultured at an air-liquid interface on ThinCertTM tissue culture inserts with 1.0 μm pores (Greiner Bio-One, Berlin, Germany) in Fitton-Jackson’s modified BGJb medium (Invitrogen, Carlsbad, CA, USA) supplemented with 5% fetal bovine serum (FBS), 100 μg/ml ascorbic acid (Invitrogen), and 100 unit/ml penicillin/streptomycin (Invitrogen).

### Mesenchyme-free tooth germ epithelium culture

E15 tooth germ rudiments were incubated in 1.4 U/ml of dispase II (Roche Diagnostics, Mannheim, Germany) in Hanks’ balanced salt solution (HBSS) at 37 °C for 20 min. Tooth germ epithelia were separated from the mesenchyme with a fine tungsten needle, and collected in 10% bovine serum albumin (BSA)/Dulbecco’s modified Eagle’s medium (DMEM)/Ham’s F12 medium. After isolated epithelia were placed in 20 μl of growth factor-reduced Matrigel (BD Biosciences, San Jose, CA, USA), the epithelia were grown in DMEM/Ham’s F12 supplemented with 1 mg/ml BSA, 1× insulin-transferrin-selenium (Life Technologies/Thermo Fisher Scientific, Waltham, MA, USA) and FGF1 (200 ng/ml; Wako Pure Chemical Industries, Osaka, Japan). When necessary, some inhibitors were added.

### Cells and antibodies

mDE6 mouse odontogenic epithelial cells and mouse mDP mouse odontogenic mesenchymal cells were kindly provided from Dr. S. Fukumoto (Tohoku University, Sendai, Japan)^53^. Lenti-X^TM^ 293 T (X293T) cells were purchased from Takara Bio Inc. (Shiga, Japan). X293T cells were grown in DMEM supplemented with 10% FBS. mDE6 and mDP cells were cultured in DMEM/F12 medium supplemented with 10% FBS. When necessary, the inhibitors, CHIR99021 (Wako), LiCl (Sigma-Aldrich, Steinheim, Germany) or SM-345431^31^, which was kindly provided from Sumitomo Dainippon Pharma Co., Ltd., cycloheximide (Wako) or trichostatin A (Wako) were added. Antibodies are listed in the Table S2.

### Microarray analyses

Microarray analyses were performed using mDE6 cells were untreated or treated with 5 μM CHIR99021 for 6 h. The mRNA expression profile was produced by Cell Innovator Inc. (Fukuoka, Japan) using gene microarray technology (SurePrint G3 Human Gene Expression Microarray 8 × 60 K v2, Agilent Technologies, Santa Clara, CA, USA). Data analyses were performed with Feature Extraction software (Agilent Technologies), and the change in ratios between the hybridization intensities of CHIR99021-treated and control samples were determined. To identify down-regulated genes, we calculated Z-scores and ratios from the normalized signal intensities of each probe. We set criteria for down-regulated genes as follows: Z-score ≦ 2.0 and ratio ≦ 0.66^54^. The raw data reported in this study was deposited in NCBI GEO under accession number (GSE116739). Functional analysis of the decreased gene expression was performed using DAVID database (http://david.abcc.ncifcrf.gov/).

### Plasmid construction and infection using lentivirus harboring a cDNA

The human Sema3A plasmid was constructed using pcDNA3.2/V5/GW/DTOPO/human Sema3A^46^. Lentiviral vector was constructed by subcloning human Sema3A cDNA into CSII-CMV-MCS-IRES2-Bsd, which was kindly provided by Dr. H. Miyoshi (RIKEN BioResource Center, Ibaraki, Japan)^55^. The vectors were then transfected along with the packaging vectors, pCAG-HIV-gp and pCMV-VSV-G-RSV-Rev, into X293T cells using the Lipofectamine LTX reagent (Invitrogen) to generate lentiviruses.

To generate mDE6 cells that stably express Sema3A for rescue experiments, parental cells (5 × 10^4^ cells/well in a 12-well plate) were treated with lentivirus and 10 μg/ml polybrene. The cells were then centrifuged at 1080 × *g* for 1 h, and incubated for another 24 h. The cells that demonstrated stable expression of Sema3A for rescue experiments, were selected and maintained in culture medium containing 5 μg/ml Blasticidin S (Wako)^56,57^.

### Knockdown of protein expression by siRNA and quantitative RT-PCR

The effects of protein knockdown by siRNA were analyzed as previously described^23,51^. Quantitative RT-PCR was performed as described previously^51^. Target sequences for siRNA and primers for quantitative RT-PCR are listed in the Table S2.

### Mutations analysis of *CTNNB1* and *APC* genes, and *Sema3A* mRNA expression in odontoma specimens

We extracted genomic DNA from paraformaldehyde-fixed paraffin-embedded (PFPE) odontoma sections using the WaxFree^TM^ Paraffin Sample DNA Extraction Kit (Trimgen, Sparks, MD, USA) according to the manufacturer’s instructions^57,58^. The mutational status of *APC* and *CTNNB1* genes was investigated using PCR and direct sequencing as described previously^59^. The PCR was performed for the mutation cluster region of the *APC* gene exon 15 (from codons 1274 to 1523) and the entire region of exon 3 of the *CTNNB1* gene, respectively. The set of primers used for these genes was the same as that previously described^59^.

Total RNA was extracted from PFPE sections using RNeasy FFPE Kit (Qiagen, Hilden, Germany)^58^. RNA was successfully obtained in only 4 odontoma specimens, because of difficulty in purification from decalcified specimens. RNA was reverse-transcribed using SuperScript VILO^TM^ (Invitrogen) in order to prepare the first-strand cDNA. Each polymerase chain reaction (PCR) product (10 µl) was directly loaded onto 2% agarose gel, stained with ethidium bromide, and directly visualized under UV illumination. Primers for RT-PCR are listed in the Table S2.

### Immunofluorescence staining

mDE6 cells, tooth germ rudiments or epithelial tooth buds were fixed for 30 min at RT in 4% PFA buffered by PBS, and then permeabilized in PBS containing 0.5% (w/v) Triton X-100 and 40 mg/ml BSA (Wako) for 30 min. The cells or rudiments were incubated with primary antibodies (used at 1:200) for 3 h at RT and then with secondary antibodies for 3 h at RT in accordance with the manufacturer’s protocols (Jackson ImmunoResearch Inc., West Grove, PA, USA). The samples were viewed and analyzed with an All-in-one Fluorescence Microscope BZ 9000 (Keyence, Osaka, Japan) and confocal microscope C2si^+^ (NIKON, Tokyo, Japan).

### Statistical analysis

Statistical analyses were performed using JMP software (SAS Institute. Inc., Cary NC, USA). Differences in results were tested for statistical significance using the Fisher exact test for Tables 1 and 2, and Student’s *t*-test for other experiments. *P* values of <0.05 were considered statistically significant.

### Additional assays

Cell proliferation and migration assays were performed as previously described^57^. Western blotting data are representative of at least three independent experiments.

## Supplementary information

Supplementary information

## Data Availability

All data generated or analyzed during this study are included in this published article and its Supplementary Information files.
