# Supplementary material for: Wnt/β-catenin signaling, which is activated in odontomas, reduces Sema3A expression to regulate odontogenic epithelial cell proliferation and tooth germ development
Source: Sci Rep. 2019 Mar 12;9:4257. doi: 10.1038/s41598-019-39686-1 (PMC6414619; doi:10.1038/s41598-019-39686-1)
Supplement: Supplementary file 1 — Supplementary information [file 41598_2019_39686_MOESM1_ESM.pdf]

Supplementary Information

Wnt/β-catenin signaling, which is activated in odontomas, reduces Sema3A expression to regulate odontogenic epithelial cell proliferation and tooth germ development

Shinsuke Fujii[1*], Kengo Nagata[1], Shinji Matsumoto[2], Ken-ichi Kohashi[3], Akira Kikuchi[2], Yoshinao Oda[3], Tamotsu Kiyoshima[1] and Naohisa Wada[4]

[1]Laboratory of Oral Pathology, Division of Maxillofacial Diagnostic and Surgical Sciences, Faculty of Dental Science, Kyushu University, 3-1-1 Maidashi, Higashi-ku, Fukuoka 812-8582, Japan

[2]Department of Molecular Biology and Biochemistry, Graduate School of Medicine, Osaka University, 2-2 Yamadaoka, Suita 565-0871, Japan

[3]Department of Anatomic Pathology, Graduate School of Medical Sciences, Kyushu University, 3-1-1 Maidashi, Higashi-ku, Fukuoka 812-8582, Japan

[4]Division of General Dentistry, Kyushu University Hospital, Kyushu University, 3-1-1 Maidashi, Higashi-ku, Fukuoka 812-8582, Japan

[*]Corresponding author. Laboratory of Oral Pathology, Division of Maxillofacial Diagnostic and Surgical Sciences, Faculty of Dental Science, Kyushu University

3-1-1 Maidashi, Higashi-ku, Fukuoka 812-8582, Japan

Phone: +81-92-642-6328; Fax: +81-92-642-6329

E-mail: sfujii@dent.kyushu-u.ac.jp

Table S1. Gene expression levels decreased after treatment with 5 μM CHIR99021 in mDE6 cells. DNA microarray analyses were performed as described in the Materials and Methods. Candidate genes were selected based on the criteria that their expression levels were lower than in control cells and then functional annotation clustering was carried out by using the DAVID database. Cluster name, gene symbol and ratio are shown.

Cluster name	Gene symbol	Ratio	Gene symbol	Ratio
Developmental protein	Bicc1	0.65	Id3	0.41
	Cited2	0.34	Kdf1	0.05
	Epha4	0.24	Lbh	0.31
	Hmx2	0.019	Mgp	0.61
	Tbx20	0.022	Mturn	0.4
	Angpt1	0.41	Ntrk3	0.29
	Atoh8	0.34	Pak3	0.53
	Ackr3	0.47	Prrx2	0.65
	Bmp4	0.58	Pdgfb	0.23
	Bmp5	0.06	Pdgfd	0.21
	Dkk3	0.58	Rhob	0.54
	Dact1	0.48	Scx	0.66
	Dlx1	0.5	**Sema3a**	**0.3**
	Eda2r	0.06	Six2	0.63
	Epas1	0.42	Six5	0.23
	Fgf17	0.05	Tshz2	0.02
	Fzd3	0.2	Vamp5	0.55
	Fzd4	0.39	Wnt5a	0.54
	Frzb	0.02	Wnt5b	0.62
	Id2	0.31	Zfp568	0.37

Cluster name	Gene symbol	Ratio	Gene symbol	Ratio
Multicellular organism	Bicc1	0.65	Kdf1	0.05
	Cited2	0.34	Lbh	0.31
	Epha4	0.24	Mgp	0.61
	Hmx2	0.019	Mmp11	0.59
	Tbx20	0.022	Mturn	0.4
	Angpt1	0.41	Ntrk3	0.29
	Atoh8	0.34	Pak3	0.53
	Ackr3	0.47	Prrx2	0.65
	Bmp4	0.58	Pdgfb	0.23
	Bmp5	0.06	Pdgfd	0.21
	Dkk3	0.58	Rhob	0.54
	Dact1	0.48	Scx	0.66
	Dlx1	0.5	**Sema3a**	**0.3**
	Eda2r	0.06	Six2	0.63
	Epas1	0.42	Six5	0.23
	Fgf17	0.05	Tshz2	0.02
	Fzd3	0.2	Vamp5	0.55
	Fzd4	0.39	Vdr	0.45
	Frzb	0.02	Wnt5a	0.54
	Id2	0.31	Wnt5b	0.62
	Id3	0.41	Zmym2	0.19

Cluster name	Gene symbol	Ratio	Gene symbol	Ratio
Differentiation	Cited2	0.65	Kdf1	0.05
	Hmx2	0.019	Lrrk2	0.5
	Rasal1	0.02	Mgp	0.61
	Anapc2	0.62	Ntrk3	0.29
	Angpt1	0.41	Rhob	0.54
	Atoh8	0.34	**Sema3a**	**0.3**
	Bmp4	0.58	Tgfb1i1	0.31

	Bmp5	0.06	Unc13a	0.27
	Eda2r	0.06	Vamp5	0.55
	Epas1	0.42	Wnt5a	0.54
	Frzb	0.02		

Cluster name	Gene symbol	Ratio
Regulation of cell growth	Wisp2	0.52
	Ctgf	0.27
	Cyr61	0.6
	Crim1	0.59
	Ddr1	0.49
	Nanos1	0.53
	Pttg1	0.59
	Sgk1	0.34

Table S2.

Antibodies used in this study.

IB, immunoblotting; IF, immunofluorescence; IHC, immunohistochemistry.

Company	Name; catalogue number	Used for
Abcam	Anti-Sema3A; ab199475	IHC, IB, IF
	Anti-Ki-67; ab16667	IHC, IF
BD Biosciences	Anti-β-catenin; 610153	IHC, IB, IF
	Anti-E-cadherin; 610181	IF
Cell Signaling Technology	Anti-Lef1; 2230	IHC, IB
	Anti-AKT; 4691S	IB
	Anti-Phospho-AKT; 4060S	IB
	Anti-ERK1/2; 4695S	IB
	Anti-Phospho-ERK1/2; 4370	IB
Sigma-Aldrich	Anti-β-actin; A5441	IB

Designs for siRNAs used in this study.

siRNA	Sequence
mouse Sema3A #1	CCTCTTGGCTTGAATATT
mouse Sema3A #2	CCAGCACTCGTGTTTGTAT
mouse β-catenin	CCACTAATGTCCAGCGCTT
mouse Lef1	CCCTGATGAAGGAAAGCAT
randomized control	CAGTCGCGTTTGCGACTGG

Forward and reverse primers for quantitative RT-PCR used in this study.

Primer	Sequence
mouse Sema3A (targeting open reading frame)	AATGGCATTGACACCCATTT
	GGGACCATCTCTGTGAGCAT
mouse Sema3A	CCTCCCAAAACCTCAAACAA

(targeting 3’-untranslated region)	TGATCTCTGTCAAGCGTTGG
mouse Axin2	CTGGCTCCAGAAGATCACAAAG
	CATCCTCCCAGATCTCCTCAAA
mouse Lef1	ACGGAGGCCTGTACAACAAG
	CTCGTCGCTGTAGGTGATGA
mouse CTNNB1	GTGCAATTCCTGAGCTGACA
	CTTAAAGATGGCCAGCAAGC
mouse cyclinD1	CACAACTTCTCGGCAGTCAA
	AGTGCGTGCAGAAGGAGATT
mouse GAPDH	GTGTCCGTCGTGGATCTGA
	TTGCTGTTGAAGTCGCAGGAG
human Sema3A	AACGGCCGTGGGAAGAGTCCAT
	TGGTGGTGCCCAAGAGTTCGG
human GAPDH	GCACCGTCAAGGCTGAGAAC
	TGGTGAAGACGCCAGTGGA

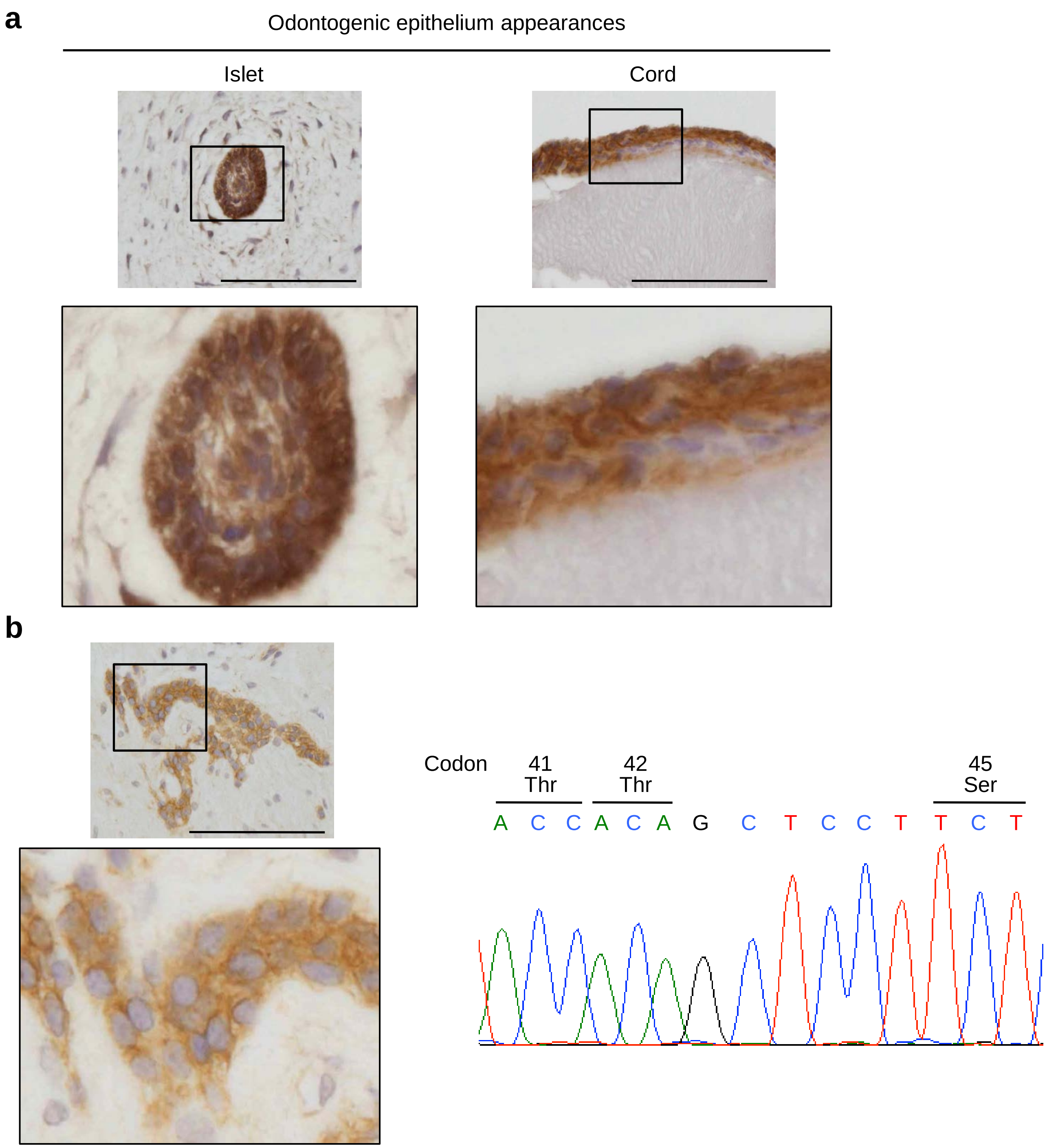

Supplementary Figure S1. Expression patterns of β-catenin in remained epithelial cells of human odontomas. (**a**) β-catenin expression is shown in remaining epithelial cells of human odontomas with islet or cord forms. Black boxes show enlarged images. (**b**) β-catenin expression is shown in remaining epithelial cells of human odontomas with nucleus/cytoplasm pattern. Genomic DNA was subjected to direct sequencing of exon 3 of the *CTNNB1* gene. Thr, threonine; Ser, serine. Scale bars: 100 μm.

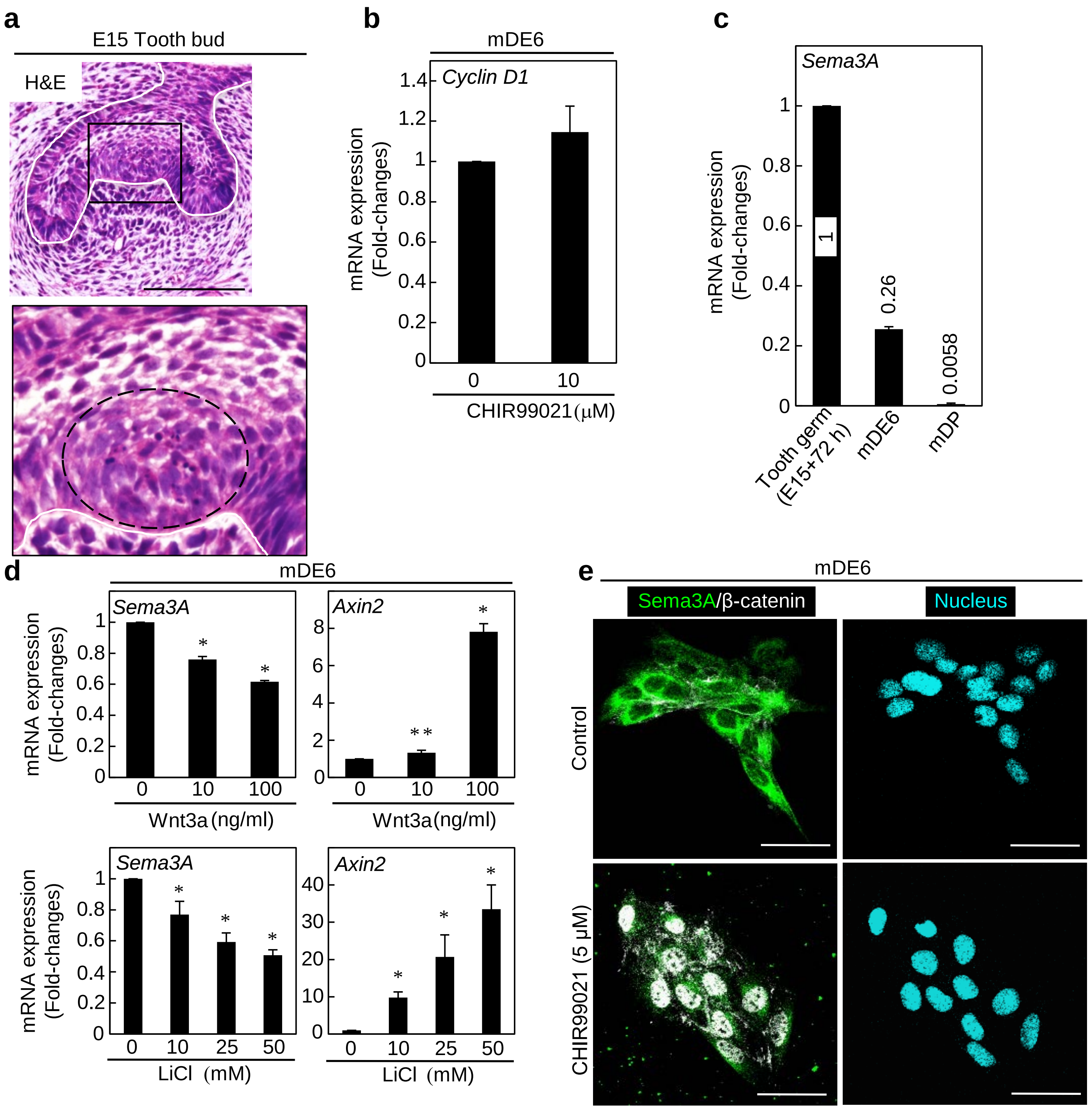

Supplementary Figure 2. Effect of Wnt signaling on mDE6.
(**a**) Sections prepared from mouse tooth germ at E15 were stained with H&E. Black boxes show enlarged images. Dotted line and white lines indicate the enamel knot region and the border between odontogenic epithelium and mesenchyme, respectively. Scale bar: 100 μm. (**b**) Expression levels of *cyclin D1* mRNA in mDE6 cells cultured without or with 10 μM CHIR99021 for 24 h were measured and represented as fold-changes compared with levels in control cells. (**c**) *Sema3A* mRNA levels in tooth germ rudiments isolated from E15 and then cultured for 72 h, mDE6 and mDP cells were measured by quantitative RT-PCR. Relative levels of *Sema3A* mRNA expression were normalized to *GAPDH* and expressed as fold-changes compared with expression in the tooth germ rudiments. (**d**) mDE6 cells were cultured without or with 10 and 100 ng/ml Wnt3a, or without or with 10, 25 and 50 mM LiCl for 24 h, and then relative levels of *Sema3A* or *Axin2* mRNA were measured and expressed as fold-changes compared with levels in control cells. (**e**) mDE6 cells were cultured without or with 5 μM CHIR99021 for 24 h, and then stained with anti-Sema3A, anti-β-catenin antibodies and Hoechst 33342. Scale bars: 50 μm. *$p < 0.01$. ** $p < 0.05$.

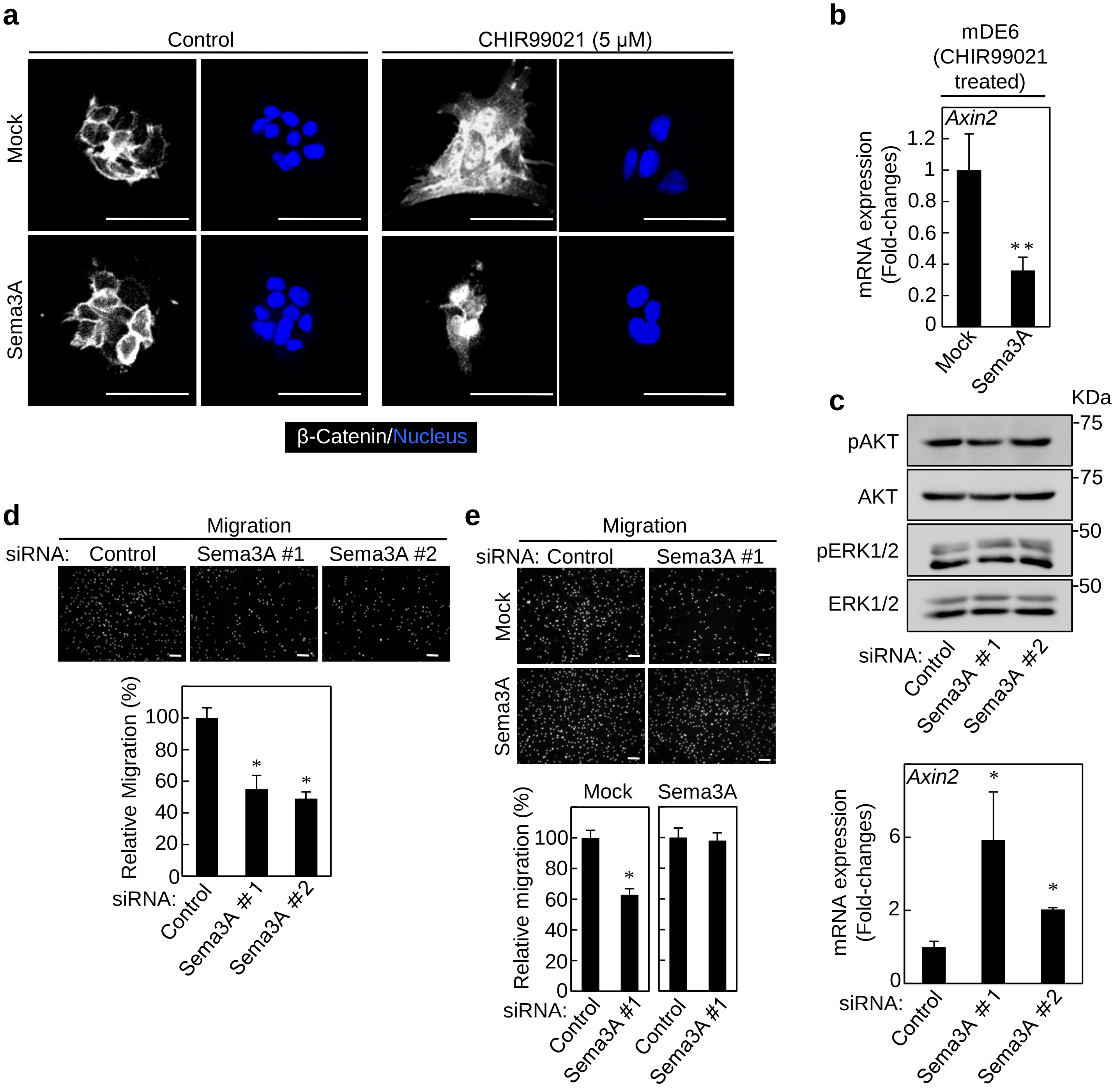

Supplementary Figure 3. Sema3A expression is involved in migration of odontogenic epithelial cells.
(**a**) mDE6 cells expressing mock or Sema3A were cultured without or with 5 μM CHIR99021 for 24 h, and then stained with anti-β-catenin antibody and Hoechst 33342. (**b**) The data was extracted from Fig. 3a. mDE6 cells expressing mock or Sema3A were cultured with 5 μM CHIR99021 for 24 h. *Axin2* mRNA levels were measured by quantitative RT-PCR. Relative *Axin2* mRNA expression levels were normalized by *GAPDH* and expressed as fold-changes compared with levels in mock cells. (**c**) mDE6 cells were transfected with control or two independent Sema3A siRNAs. Cell lysates were probed with anti-phospho-AKT, anti-AKT, anti-phospho-ERK1/2 and anti-ERK1/2 antibodies. *Axin2* mRNA levels were measured by quantitative RT-PCR. Relative *Axin2* mRNA expression levels were normalized by *GAPDH* and expressed as fold-changes compared with levels in control siRNA transfected mock cells. (**d**) mDE6 cells were placed in Transwell chamber for the migration assay. Migration activities are expressed as the percentage of migrated cell number observed in control cells. (**e**) mDE6 cells expressing mock or Sema3A were transfected with control or Sema3A #1 siRNA. The cells were placed in Transwell chamber for the migration assay. Migration activities are expressed as the percentage of migrated cell number observed in control cells. Results are shown as means ± s.d. of three independent experiments. *$p < 0.01$. **$p < 0.05$. Scale bars: 50 μm (a), 200 μm (d-e).

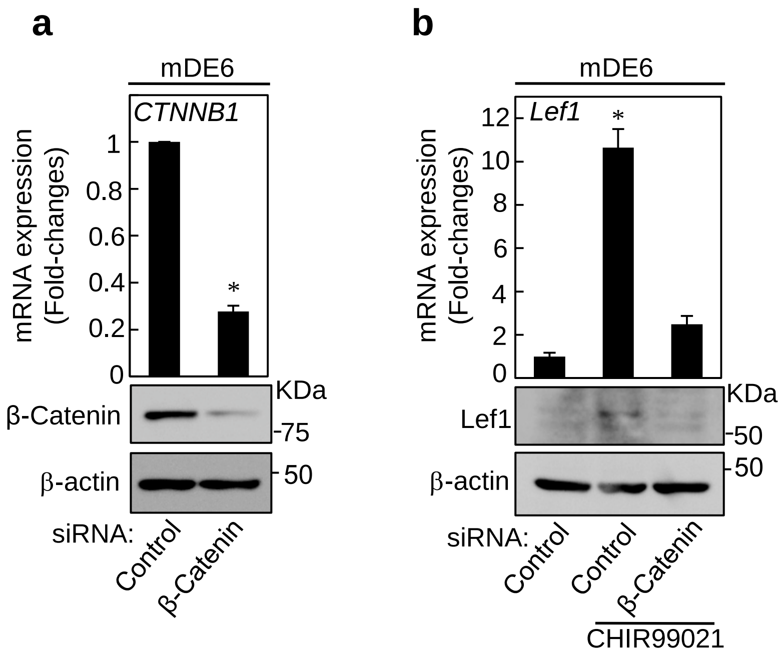

Supplementary Figure 4. Wnt/β-catenin-Lef1 signaling downregulates Sema3A expression.
(**a**) mDE6 cells were transfected with control or β-catenin siRNAs, and *CTNNB1* mRNA levels were measured by quantitative RT-PCR. Relative *CTNNB1* mRNA levels were normalized by *GAPDH* and expressed as fold-changes compared with levels in control siRNA transfected cells. Cell lysates were probed with anti-β-catenin and anti-β-actin antibodies. (**b**) mDE6 cells were transfected with control or β-catenin siRNA, and then were cultured with 10 μM CHIR99021 for last 12 h. Relative *Lef1* mRNA levels were normalized by *GAPDH* and expressed as fold-changes compared with levels in control siRNA transfected cells. Cell lysates were probed with anti-Lef1 and anti-β-actin antibodies. (**c**) mDE6 cells were transfected with control or Lef1 siRNA, and *Lef1* mRNA levels were measured by quantitative RT-PCR. Relative *Lef1* mRNA levels were normalized by *GAPDH* and expressed as fold-changes compared with levels in control siRNA transfected cells. Results are shown as means ± s.d. of three independent experiments. * $p < 0.01$.

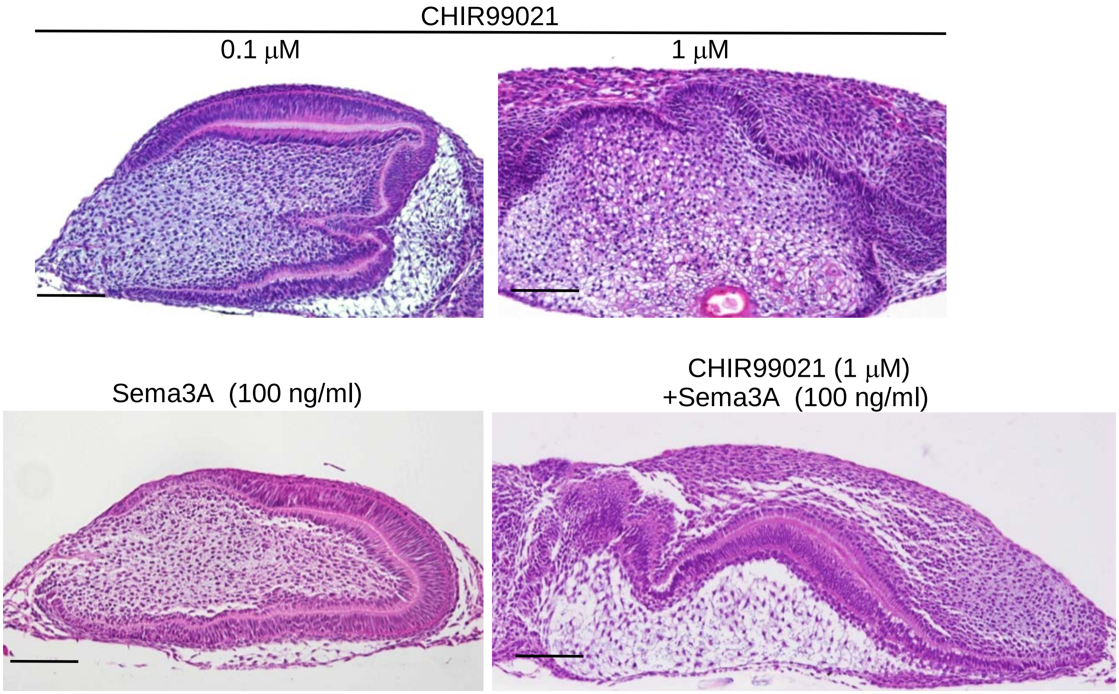

Supplementary Fig. 5. Effect of Wnt signaling on tooth germ development.
Tooth germ rudiments were cultured without or with 0.1 and 1 μM CHIR99021, 100 ng/ml Sema3A or 1 μM CHIR99021 with 100 ng/ml Sema3A for 7 days, and then the rudiments were stained with H&E. Scale bars: 100 μm.

a (Figure 2d)

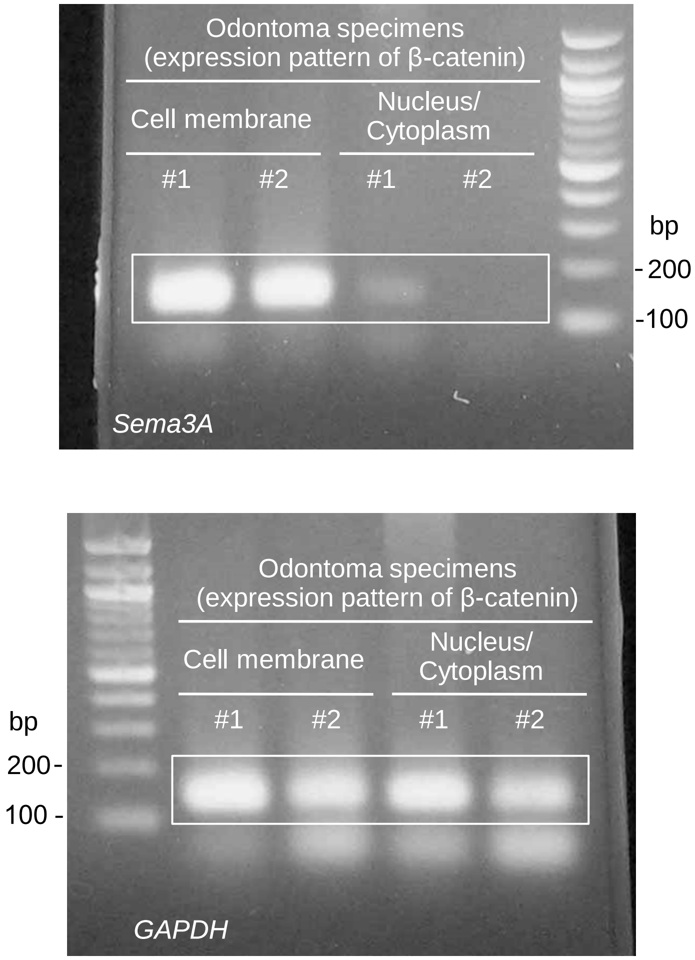

b (Figure 2e)

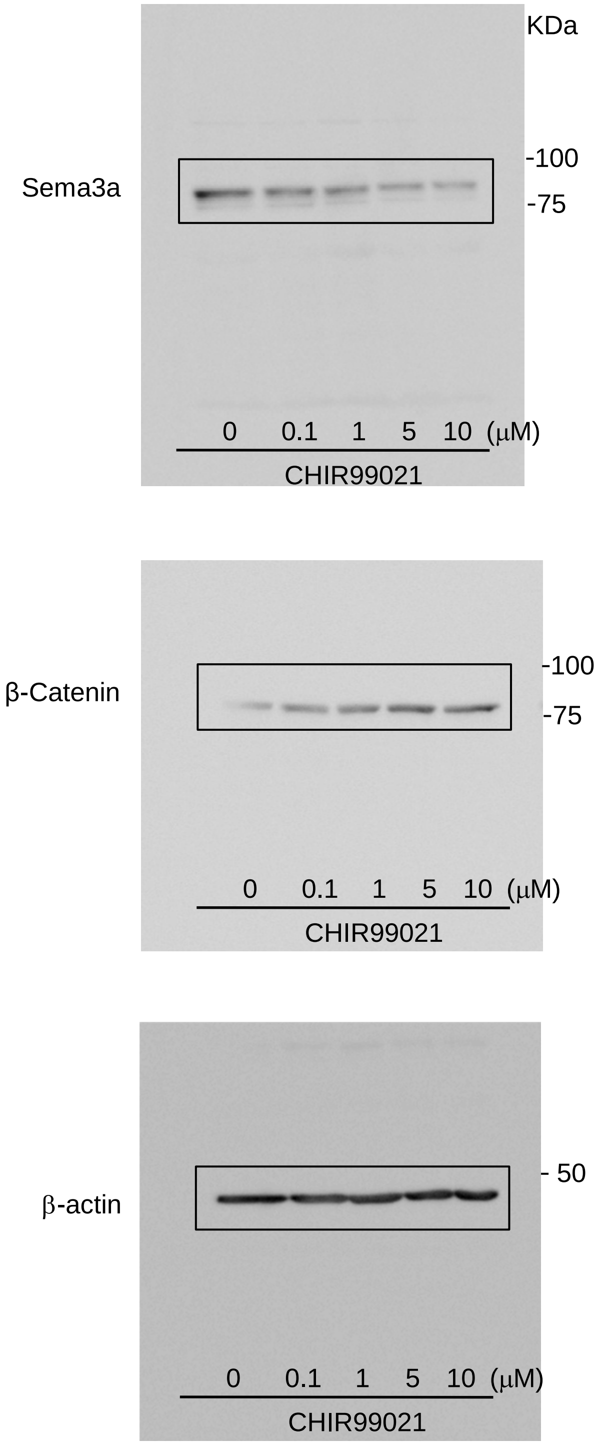

Supplementary Fig. 6. Full scan images of electrophoresis and immunoblots presented in Figure 2.

a (Figure 3a)

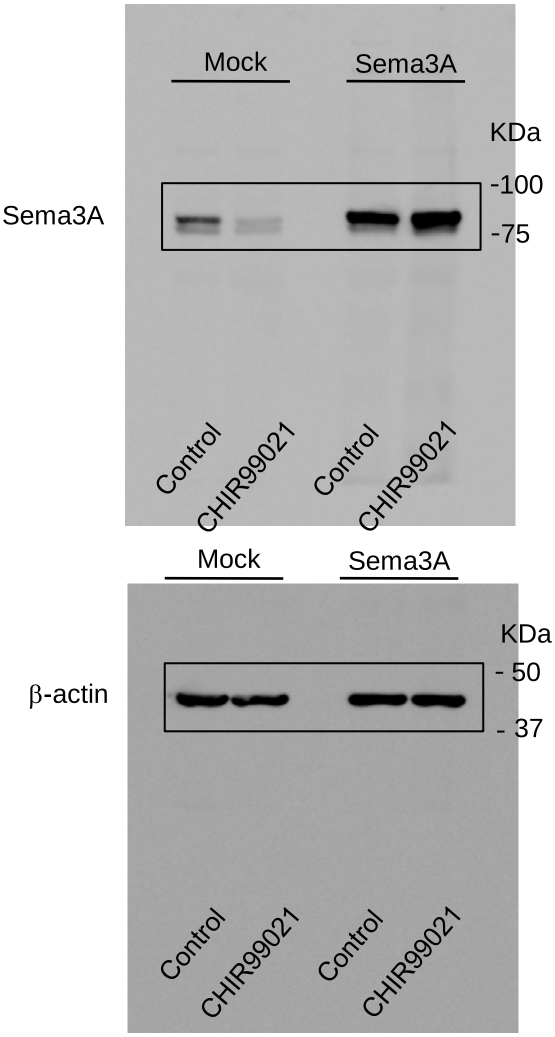

b (Figure 3c)

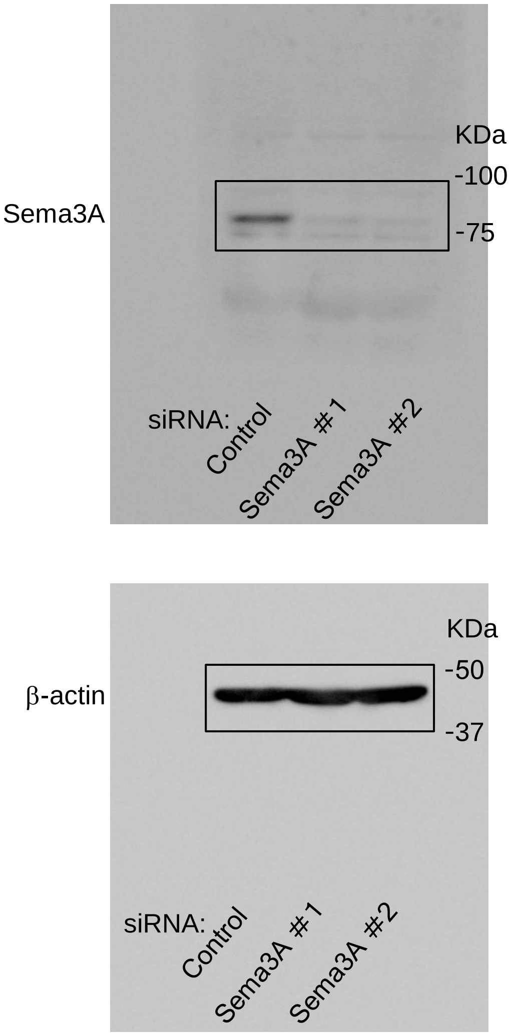

c (Figure 3e)

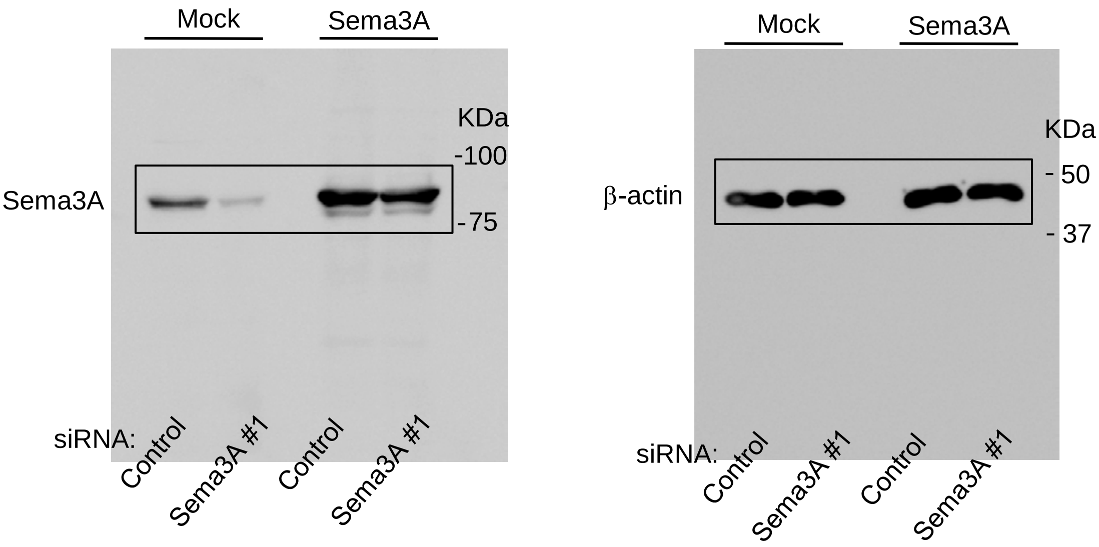

Supplementary Fig. 7. Full scan images of immunoblots presented in Figure 3.

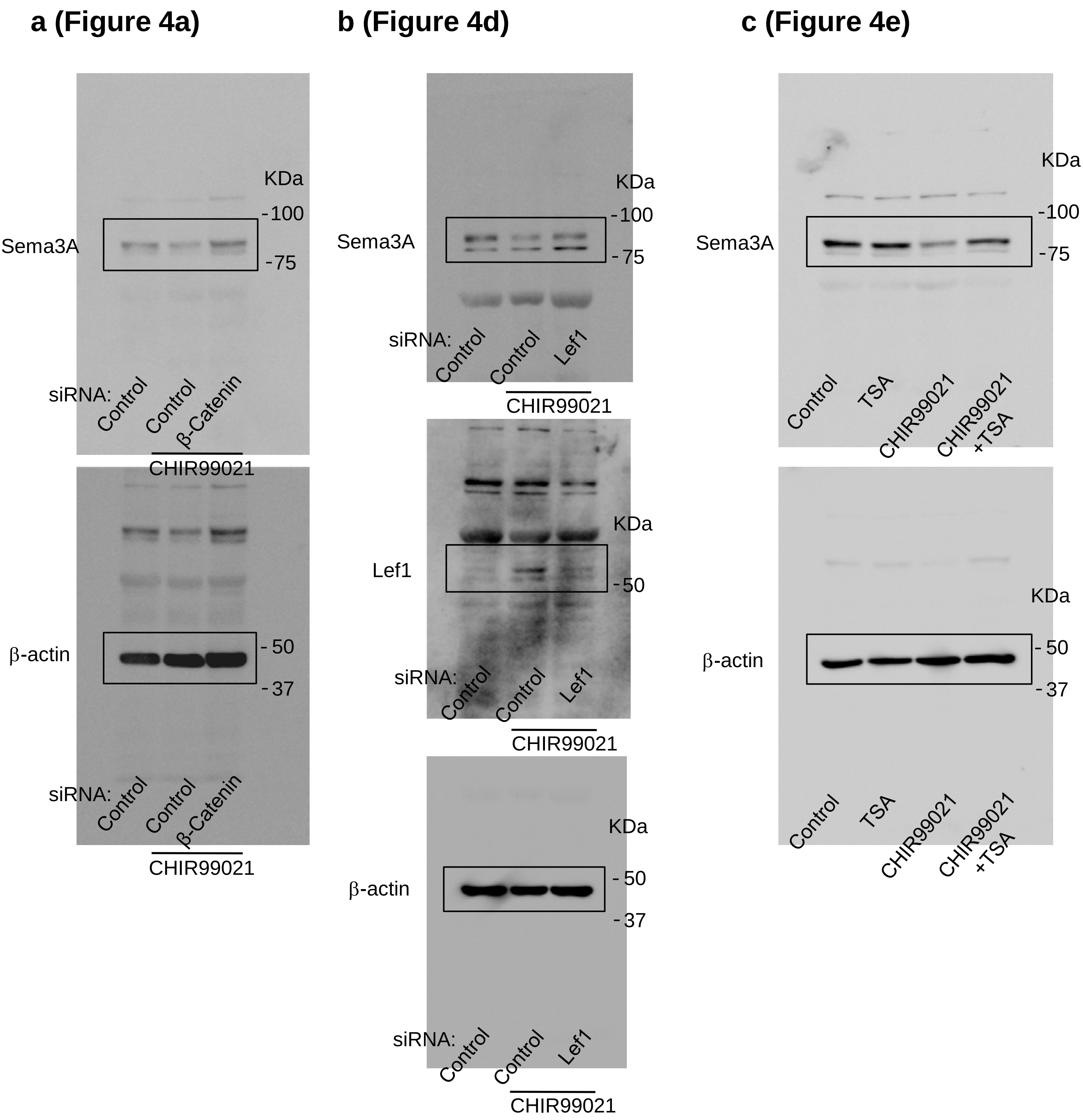

Supplementary Fig. 8. Full scan images of immunoblots presented in Figure 4.

(Figure S3c)

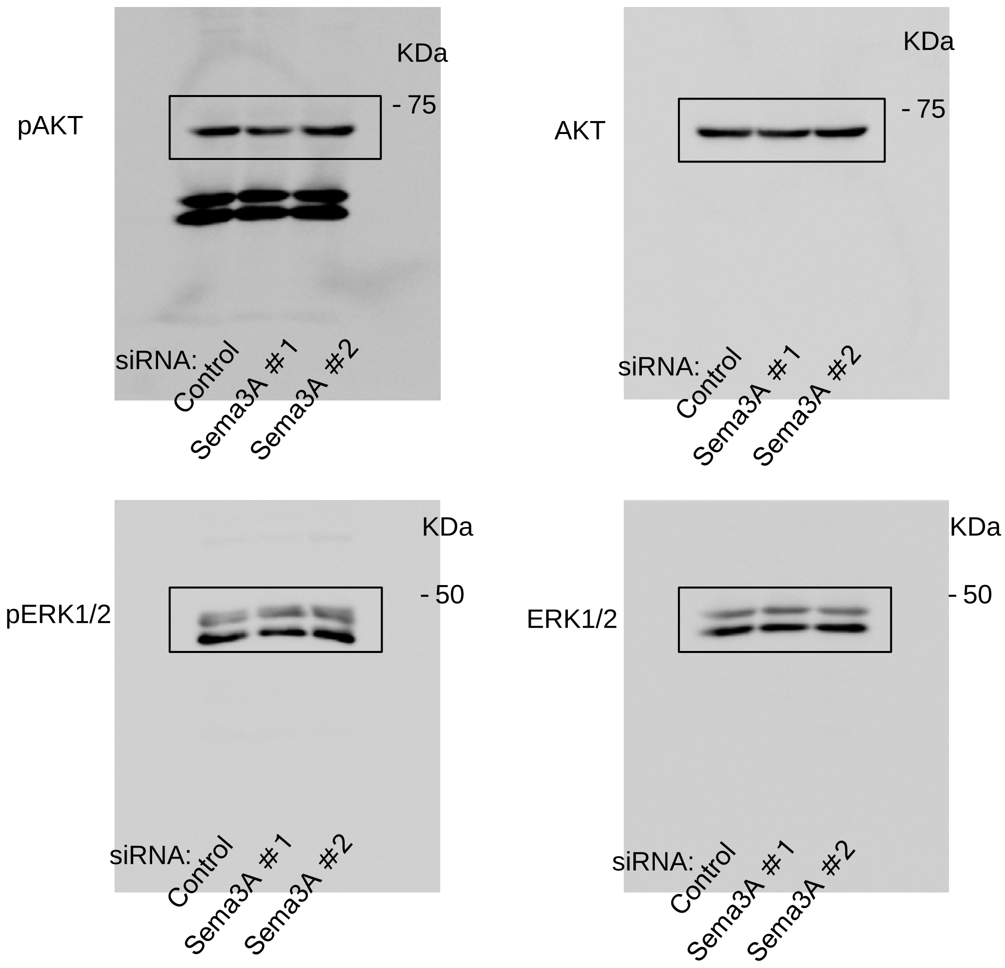

Supplementary Fig. 9. Full scan images of immunoblots presented in Figure S3.

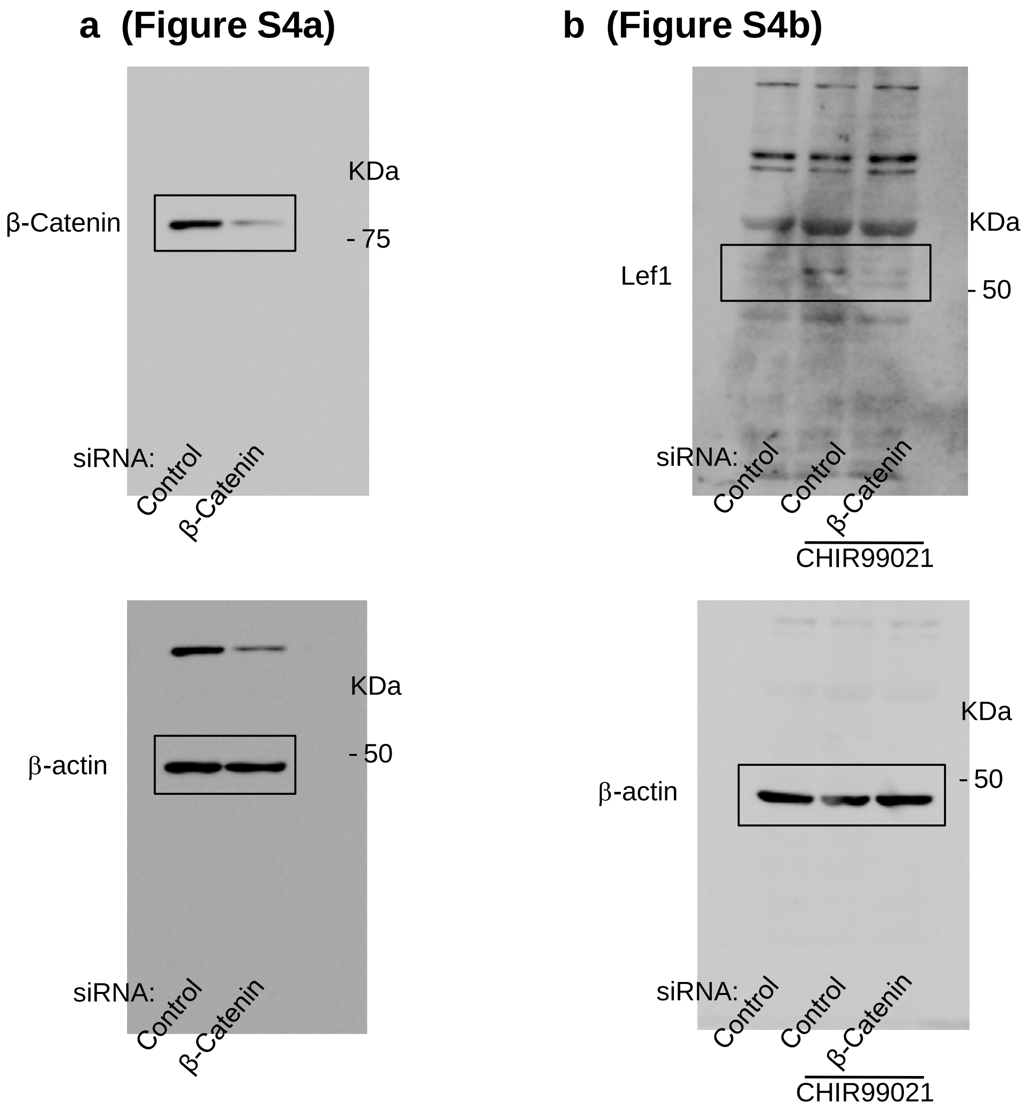

Supplementary Fig. 10. Full scan images of immunoblots presented in Figure S4.
